# Comparison of clinical performance of antigen based-enzyme immunoassay (EIA) and major outer membrane protein (MOMP)-PCR for detection of genital Chlamydia trachomatis infection 

**Published:** 2016-06

**Authors:** Mahmoud Nateghi Rostami, Batool Hossein Rashidi, Fatemeh Aghsaghloo, Razieh Nazari

**Affiliations:** 1 *Department of Microbiology and Immunology, Faculty of Medicine, Qom University of Medical Sciences, Qom, Iran.*; 2 *Department of Obstetrics and Gynecology, Vali-Asr Reproductive Health Research Center, Tehran University of Medical Sciences, Tehran, Iran.*; 3 *Department of Microbiology, Islamic Azad University, Qom Branch, Qom, Iran.*

**Keywords:** *Chlamydia trachomatis*, *Pregnancy complications*, *Enzyme immunoassay*, *Nested PCR*

## Abstract

**Background:**

*Chlamydia trachomatis* is the most common sexually transmitted bacterial pathogen worldwide. Early detection and treatment of *C.trachomatis* genital infection prevent serious reproductive complications.

**Objective:**

Performances of enzyme immunoassay (EIA) and major outer membrane protein (MOMP)-polymerase chain reaction (PCR) for diagnosis of genital *C.trachomatis* infection in women were compared.

**Materials and Methods:**

In this cross sectional study a total of 518 women volunteers were included (33.67±8.3 yrs) who had been referred to Gynecology clinics of Qom province, Iran, were included. Endocervical swab specimens were collected to detect lipopolysaccharide (LPS) antigen in EIA and to amplify MOMP gene of* C.trachomatis *in PCR. Results were confirmed using *ompI* nested-PCR. Sensitivity, specificity, positive (PPV) and negative predictive values (NPV) were calculated for performance of the tests. Odds ratios were determined using binary logistic regression analysis.

**Results:**

In total, 37 (7.14%) cases were positive by EIA and/or MOMP-PCR. All discrepant results were confirmed by nested-PCR. Sensitivity, specificity, PPV and NPV values of EIA were 59.46%, 100%, 100% and 96.98%, and those of MOMP-PCR were 97.30%, 100%, 100%, 99.79%, respectively. Reproductive complications including 2.7% ectopic pregnancy, 5.4% stillbirth, 5.4% infertility, and 10.8% PROM were recorded. The risk of developing chlamydiosis was increased 4.8-fold in volunteers with cervicitis (p<0.05; OR 4.80; 95% CI 1.25-18.48).

**Conclusion:**

*C.trachomatis *infection should be regarded in women of reproductive ages especially those with cervicitis. Primary screening of women by using the low cost antigen-EIA is recommended; however, due to the low sensitivity of Ag-EIA, verification of the negative results by a DNA amplification method is needed.

## Introduction

According to World Health Organization (WHO) 350-500 million new cases of curable sexually transmitted infections (STIs), including *Treponema pallidum*, *Neisseria gonorrhoeae, Chlamydia trachomatis (C.trachomatis) *and *Trichomonas vaginalis*, occur annually throughout the world. More than 1 million people acquire a STI every day, with the largest proportion in the south and south-east Asia. More than 30 bacterial, viral and parasitic pathogens are causative agents of STIs. Currently *C.trachomatis* is the most common sexually transmitted bacterial pathogen worldwide (1). *C.trachomatis* is an obligate intracellular bacterium that infects mucosal surfaces of the cervix, urethra, rectum, nasopharynx, and conjunctiva. Genital chlamydiosis is the most frequently reported sexually transmitted disease (STD) in the United States with an increasing rate of infection more commonly in women than in men which was reported in the last 5 years(2). 

In Iran, the estimated prevalence of *Chlamydia* infection seems to be more than 10% in general population, and up to 30% in specific high-risk groups, such as sex workers (3). High-risk sexual behaviors, low socioeconomic levels, young age, specific symptoms such as mucopurulent discharge are among important risk factors for chlamydial infection (4-7). The complications of chlamydial infection in women include the facilitation of HIV transmission, ocular infections of the newborn, disseminated infection, ectopic pregnancy, preterm labor, and pelvic inflammatory diseases (PIDs) which is one of the major causes of infertility (8, 9). However, *C. trachomatis* genital infections are often asymptomatic in women. Early detection of *C. trachomatis* genital infection to avoid serious complications would be valuable since effective treatment is available and with prompt antibiotic treatment most of these complications might be prevented. 

The Center for Disease Control and Prevention recommends *Chlamydia* screening for all sexually active women less than 26 years old. Evidences showed that in areas where the screening programs were implemented, the prevalence of infection and rate of PIDs were decreased (2). Traditionally, the gold standard for the identification of *C. trachomatis* infections is tissue culture, which is a time consuming and labor-intensive method and requires trained staff. Common serological techniques in the diagnosis of *Chlamydia *antibodies have some pitfalls, including cross reactivities and long persistence of IgG antibody even after treatment and cure (10, 11). 

In recent years, several nucleic acid amplification techniques and polymerase chain reaction (PCR)-based methods have become available which provide improved sensitivity for diagnosis of *C. trachomatis* infection. Although different molecular amplification tests are increasingly developed, their use as routine screening tests for *C. trachomatis* is limited by the high cost and need of facilities. Antigen-based EIA might be an affordable alternative since it is less technically complex and less costly compared to DNA amplification techniques and provides more accuracy than antibody detection using serological techniques.

Since there have been no previous study dealing with chlamydial detection by antigen-EIA in Iran, in this study performance of Ag-EIA and conventional PCR in detection of *C. trachomatis* infection in endocervical swab specimens is reported.

## Materials and methods

**Study population, ethical considerations and sampling**

The study protocol conforms to the ethical guidelines of the 1975 Declaration of Helsinki and has been approved by Ethical Committee of Qom University of Medical Sciences. A written informed consent was obtained from each volunteer. In this cross sectional study, from May 2013 to April 2014, a total of 518 women who admitted to gynecology clinics of Qom province, Iran, for Pap smear or regular checkup were randomly recruited. 

Inclusion criteria were age range between 18-50 years and no administration of systemic or topical antibiotics during last one month. Each volunteer was examined by gynecologist and demographic data, history and clinical signs were recorded in a questionnaire form. Swab samples were collected from endocervical canal under aseptic conditions. First swab was placed in a tube containing sterile PBS and stored at 4ºC until use for EIA; and the second swab was transferred to sterile PBS, pH=7.4, and kept at -20ºC for PCR. 

**EIA for detection of** ***Chlamydia trachomatis*** **antigen**

The level of *C. trachomatis* antigen was measured by EIA method using MicroTrak^®^II *Chlamydia* EIA kit (Trinity Biotech, Ireland).Specimen treatment solution was used to elute lipopolysaccharide (LPS) antigen from the genital swabs in the assay procedure. After adding 1 ml of specimen treatment solution to patient swabs, specimens and controls tubes were heated in a preheated block (100^o^C) for 15 min. The microwell plates were coated with 100 µl of rabbit chlamydial anti-LPS mAb in PBS, pH=7.2, and 100 µl of genital discharge samples or controls were added and plates were incubated for 90 min at 37^o^C. 

Next, 100 µl of peroxidase-labeled anti-rabbit IgG was added and plates were incubated at 37^o^C for 30 min. 100 µl of 3,3’,5,5’-tetramethyl benzidine (TMB) substrate (Sigma, St. Louis, MO, USA) was added and plates were incubated at 37^o^C for 30 min and then the reaction was stopped with 100 µl of 0.5 M H_2_SO_4_ solution (Merck, Darmstadt, Germany).

The plates were washed after each step of incubation using PBS+0.05% (v/v) Tween 20 (Sigma, St. Louis, MO, USA). The plates were read at 450 nm using a reader (BioTek, Winooski, VT, USA). The mean optical densities (ODs) of triplicate samples represent the differences between the ODs of test and background wells. Cutoff value was determined by adding the mean of negative control absorbance values to a constant. Samples with absorbance’s equal to or greater than the cutoff value were considered positives. Based on the information provided with the kit, common genital organisms like *Candida* spp., *T.vaginalis*, *Neisseria* spp., Enterobacteriaceae family, and Gram positive cocci have absorbance values below the cutoff. 

**DNA extraction**

DNA extraction was carried out on the endocervical samples by using AccuPrep® Genomic DNA Extraction Kit (BIONEER, South Korea). Briefly, samples in 1.5 ml tubes were centrifuged 5 min at 7000 rpm, then 20 µl of proteinase K was added to the pellet followed by adding 200 µl of GC binding buffer, tubes were incubated at 60^o^C water bath for 10 min. Then 100 µl of absolute isopropanol was added and the contents were transferred to Binding tubes. Tubes were washed with 500 µl of W1 washing buffer at 8000 rpm for 1 min, then with W2 washing buffer at 12000 rpm for 1 min. Finally, 50 µl of Elution buffer (EL) was added to the tubes and centrifuged at 8000 rpm for 1 min and elutes were collected as DNA and kept at -20^o^C until use.

**MOMP-based PCR**

For identification of *C. trachomatis, *major outer membrane protein (MOMP) target gene of the DNA samples was amplified using primers Ch-F 5'-CCTgTggggAATCCTgCTgAA -3' and Ch-R 5'-gTCgAAAACAAAgTCACC ATAgTA-3', yielding a 240 bp fragment (CinnaGene, Tehran, Iran). Amplification of the target gene was carried out in a total volume of 25 µl containing 2.5 µl 10× PCR buffer, 1.5 mM MgCl_2_, 0.5 µl of each primer (25 pmol/ml), 0.2 mM of each dNTPs, 0.5 U *Taq* DNA polymerase (all from Fermentas Life Sciences, York, UK) and 1 μM (1-3 μL) of DNA. The cycling program on thermal cycler (Eppendorf Mastercycler, Germany) was initiated by early denaturation of 5 min at 95^o^C, followed by 35 cycles of 94^o^C for 60 sec, 58^o^C for 60 sec, and 72^o^C for 60 sec, then by a final extension step at 72^o^C for 7 min. PCR products were electrophoresed on 1.5% agarose gel and visualized by staining with ethidium bromide under a UV Gel Documentation System (UVItec, Cambridge, UK). 

***ompI*** **gene** **nested-PCR**

*C.trachomatis* *ompI *gene was targeted in nested-PCR amplification. The primers used for generating an approximately 1100 bp fragment of the *ompI* gene were Ex-p1: 5'-ATgAAAAAACTCTTgAAATCgg-3' and Ex-p2: 5'-TTTCTAgATTTTCATCTTgTT-3' (Cinna Gene, Tehran, Iran). The primary PCR reaction was performed in a final volume of 50 µl containing 10 µl of DNA, 0.125 µl of each 200 pm external primers, 0.5 µl of 5 µM/µl *Taq* DNA polymerase, 5 µl of 25 mM MgCl_2_, 0.8 µl of 12.5 mM dNTPs, and 5 µl of 10× PCR buffer (all from Fermentas Life Sciences, York, UK). 

The PCR amplification on Eppendorf Mastercycler, was started with 10 min of denaturation at 95^o^C and continued with 49 cycles of amplification consisted of a denaturation step at 95^o^C for 1 min, an annealing step at 48^o^C for 3 min, and extension step at 72^o^C for 3 min. Then, 10 µl of a 1:10 dilution in distilled water of the primary PCR product was used for nested-PCR. The reaction was performed in a final volume of 50 µl containing the same PCR reaction mixture except that primers sequences were as follows: In-p1 5'-TTTCTAgATTTTCATCTTgTT-3' and In-p2 5'-TCCTTgCAAgCTCTgCCTgTggggAATCCT-3' as nested PCR primers (CinnaGene, Tehran, Iran). The amplification condition was the same as that of the primary *ompI* PCR. The primers generate an approximately 1000 bp fragment. The PCR products were analyzed by 1.5% agarose gel electrophoresis and visualized by staining with ethidium bromide under a UV Gel Documentation System (UVItec, Cambridge, UK).

**Statistical analysis**

Statistical analysis was performed by using SPSS version 18 (SPSS Inc., Chicago, IL, USA) and GraphPad Prism version 5.01 (GraphPad Software Inc., La Jolla, CA, USA) softwares. Pearson ^2^ and Fisher’s exact tests were used for the different comparisons. Using univariate logistic regression analysis odds ratios (OR) of different risk factors were calculated. Sensitivity, specificity, positive predictive value (PPV) and negative predictive value (NPV) were calculated for reporting performance of the tests. *P*≤0.05 was considered as significant. 

## Results

The mean±SD age of the women was 33.67±8.30 years, and most of the women were assigned to the 30-39 years age group, with 38% of the total recorded volunteers. As little as 3.5% of the volunteers were suffering from noncommunicable chronic diseases such as diabetes. More than 80% of the volunteers chose at least one of the contraceptive methods, including natural contraception as the most frequent method. About half of the volunteers had malodorous discharge, and about 40% had itching and dyspareunia, and history of abortion was seen in approximately 25% of the volunteers. Other complications such as ectopic pregnancy, prematurity, and infertility also were seen in the volunteers. 

**Figure 1:**
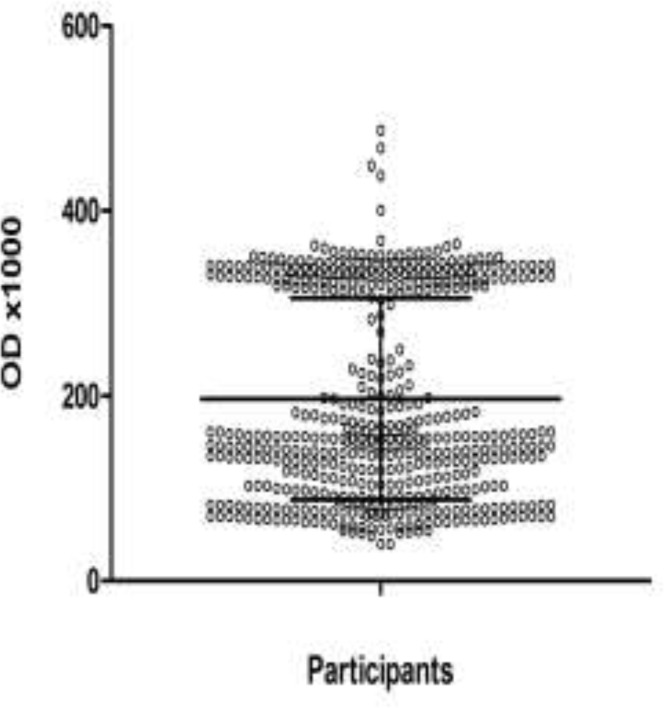
Scatter plot showing the ODs of samples in enzyme immune assay (EIA) for detection of *C. trachomatis* antigen (Resolution 300×300 dpi

**Figure 2:**
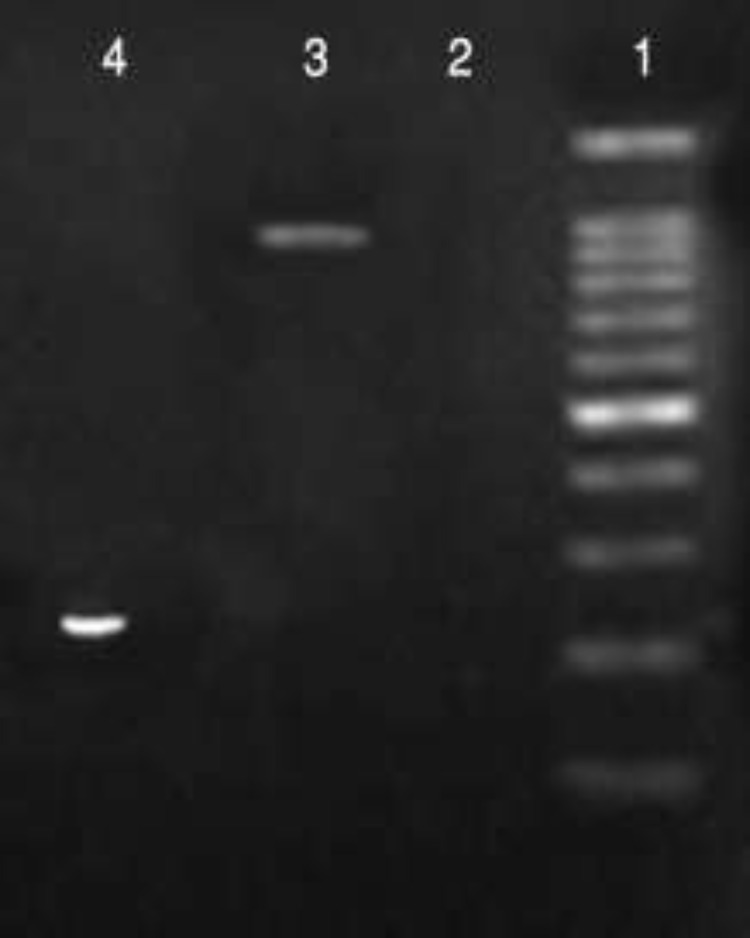
Electrophoresis of the amplification products of *Chlamydia* MOMP PCR and *ompI* nested-PCR (Resolution 300×300 dpi)Lane 1: 100 bp ladder; Lane 2: negative control; Lane 3: 1000 bp nested-PCR product; Lane 4: 240 bp MOMP PCR product

In EIA the cutoff value was determined 0.345. OD values between 0.3-0.344 were considered as suspected results and the samples were re-examined (Figure 1). Among 518 volunteers, 37 (7.14%) have EIA and/or MOMP PCR positive results which considered as chlamydiosis, and 481 (92.86%) have negative results which considered as non-chlamydiosis cases. All positive EIA samples, except one, showed specific MOMP band in analysis with PCR method (Figure 2). Positive results of EIA and all discrepant results were confirmed by nested-PCR (Figure 2). 

As it is shown in table I, one specimen with a false-negative PCR result was positive in nested-PCR assay. Fifteen of PCR-positive, EIA negative specimens were reamplified and analysed by using nested-PCR and the results remained positive. Sensitivity, specificity, PPV and NPV of EIA were 59.46% (95% confidence interval (CI) 42.10% to 75.23%), 100% (95% CI 99.23% to 100%), 100% (95% CI 84.43% to 100%) and 96.98% (95% CI 95.06% to 98.30%), respectively. Sensitivity, specificity, PPV and NPV of MOMP PCR were 97.30% (95% CI 85.79% to 99.55%), 100% (95% CI 97.87% to 100%), 100% (95% CI 90.17% to 100%), and 99.79% (95% CI 98.84% to 99.97%), respectively.

Among the positive chlamydiosis patients, 13.5% had cervicitis, 37.8% had malodorous discharge, and 48.6% had itching. In addition, 2.7% reported ectopic pregnancy, 5.4% stillbirth, 5.4% infertility, 10.8% PROM and 10.8% curettage. Pearson Chi-square analysis showed a significant difference between chlamydiosis and non-chlamydiosis volunteers with regard to cervicitis (p=0.008). Univariate logistic regression analysis showed that the risk of developing chlamydiosis was increased 4.8-fold in the volunteers with cervicitis (Table II) (p<0.05; OR 4.80; 95% CI 1.25-18.48). 

Using backward-Wald analysis, cervicitis remained an independent risk factor for the development of chlamydiosis (p=0.018; OR 4.75; 95% CI 1.08-18.13). The risk of developing chlamydiosis was decreased in the volunteers with low abdominal pain (p<0.05; OR 0.32; 95% CI 0.13-0.82).

**Table I:** Comparison of the results of EIA and MOMP PCR analysis

**Test**				**PCR**		**Total**
				**Positive**	**Negative**	
EIA						
	Positive			16 (3.0)	1 (0.2)	17 (3.3)
	Negative			21 (4.1)	480 (92.7)	501 (96.7)
Total				37 (7.1)	481 (92.9)	518 (100.0)

Data are presented as n(%).

**Table II:** Results of regression analysis in two women groups categorized based on genital *C. trachomatis* infection

**Characteristics**		**Women number (%)**			**p-value**	**OR**	**95% CI**
		**Non-chlamydiosis**	**Chlamydiosis**	**Total**			
Pregnancy							
	No *	474 (98.5%)	37 (100.0%)	511 (98.6%)	--------	--------	--------
	Yes	7 (1.5%)	0 (0.0%)	7 (1.4%)	0.999	0.000	0.000
Contraception							
	No *	79 (16.4%)	6 (16.2%)	85 (16.4%)	--------	--------	--------
	Yes	402 (83.6%)	31 (83.8%)	433 (83.6%)	0.974	0.985	0.398-2.439
Method of contraception							
	Condom *	84 (17.5%)	8 (21.6%)	92 (21.2%)	--------	--------	--------
	Natural family planning	142 (29.5%)	9 (24.3%)	151 (34.9%)	0.588	1.133	0.721-1.781
	IUD	44 (9.1%)	0 (0.0%)	44 (10.2%)	0.398	1.198	0.788-1.821
	OCP	34 (7.1%)	4 (10.8%)	38 (8.8%)	0.130	0.700	0.441-1.111
	Vasectomy	23 (4.8%)	1 (2.7%)	24 (5.5%)	0.053	1.516	0.958-2.172
	Tubectomy	59 (12.3%)	4 (10.8%)	63 (14.5%)	0.588	1.133	0.721-1.781
	Contraceptive injection	6 (1.2%)	2 (5.4%)	13 (3.0%)	0.398	1.198	0.788-1.821
	Combination	10 (2.1%)	3 (8.1%)	8 (1.9%)	0.130	0.700	0.441-1.111
	None	79 (16.4%)	6 (16.2%)	85 (16.4%)	0.053	1.516	0.958-2.172
Antibiotic administration							
	No *	377 (78.4%)	29 (78.4%)	406 (80.4%)	--------	--------	--------
	Yes	104 (21.6%)	8 (21.6%)	112 (19.6%)	0.986	0.991	0.359-2.739
History of chronic diseases							
	No *	463 (96.3%)	37 (100.0%)	500 (96.5%)	--------	-------	---------
	Yes	18 (3.7%)	0 (0.0%)	18 (3.5%)	0.999	0.000	0.000
Number of kids							
	0 *	60 (12.5%)	6 (16.2%)	66 (12.7%)	--------	--------	--------
	1-2	250 (52.0%)	21 (56.8%)	271 (52.3%)	0.719	0.840	0.325-2.172
	3-4	124 (25.8%)	10 (27.0%)	134 (25.9%)	0.690	0.806	0.280-2.323
	≥ 5	47 (9.8%)	0 (0.0%)	47 (9.1%)	0.997	0.000	0.000
Dysuria							
	No *	455 (94.6%)	34 (91.9%)	489 (94.4%)	--------	-------	---------
	Yes	26 (5.4%)	3 (8.1%)	29 (5.6%)	0.595	0.552	0.062-4.948
Malodorous discharge							
	No *	264 (54.9%)	23 (62.2%)	287 (55.4%)	--------	-------	---------
	Yes	217 (45.1%)	14 (37.8%)	231 (44.6%)	0.798	0.884	0.345-2.267
Cervicitis							
	No *	462 (96.0%)	32 (86.5%)	494 (95.4%)	--------	-------	-----------
	Yes	19 (4.0%)	5 (13.5%)	24 (4.6%)	0.022	4.803	1.250-18.482
Low abdominal pain							
	No *	221 (45.9%)	22 (59.5%)	243 (46.9%)	--------	-------	-----------
	Yes	260 (54.1%)	15 (40.5%)	275 (53.1%)	0.018	0.323	0.127-0.822
Dyspareunia							
	No *	280 (58.2%)	22 (59.5%)	302 (58.3%)	--------	-------	------------
	Yes	201 (41.8%)	15 (40.5%)	216 (41.7%)	0.064	1.247	0.497-3.130
Itching							
	No *	302 (62.8%)	19 (51.4%)	321 (62.0%)	--------	-------	------------
	Yes	179 (37.2%)	18 (48.6%)	197 (38.0%)	0.416	1.457	0.589-3.609
Abortion							
	No *	360 (74.8%)	30 (81.1%)	390 (75.3%)	--------	-------	------------
	Yes	121 (25.2%)	7 (18.9%)	128 (24.7%)	0.366	0.546	0.147-2.024
Ectopic pregnancy							
	No *	474 (98.5%)	36 (97.3%)	510 (98.5%)	--------	-------	------------
	Yes	7 (1.5%)	1 (2.7%)	8 (1.5%)	0.730	1.561	0.124-19.665
Stillbirth							
	No *	459 (95.4%)	35 (94.6%)	494 (95.4%)	--------	-------	------------
	Yes	22 (4.6%)	2 (5.4%)	24 (4.6%)	0.267	2.712	0.467-15.755
Infertility							
	No *	439 (91.3%)	35 (94.6%)	474 (91.5%)	--------	-------	------------
	Yes	42 (8.7%)	2 (5.4%)	44 (8.5%)	0.874	1.230	0.095-15.907
Prematurity							
	No *	467 (97.1%)	36 (97.3%)	503 (97.1%)	--------	-------	------------
	Yes	14 (2.9%)	1 (2.7%)	15 (2.9%)	0.801	1.396	0.104-18.747
	PROM						
	No *	452 (94.0%)	33 (89.2%)	485 (93.6%)	--------	-------	-----------
	Yes	29 (6.0%)	4 (10.8%)	33 (6.4%)	0.065	3.121	0.933-11.693
Curettage							
	No *	412 (85.7%)	33 (89.2%)	445 (85.9%)	--------	-------	------------
	Yes	69 (14.3%)	4 (10.8%)	73 (14.1%)	0.753	0.740	0.114-4.809
Age (years)							
	15-19	11 (2.3%)	1 (2.7%)	12 (2.3%)	--------	--------	--------
	20-29	157 (32.6%)	11 (29.7%)	168 (32.5%)	0.691	1.118	0.643-1.944
	30-39	184 (38.3%)	13 (35.1%)	197 (38.0%)	0.744	0.908	0.510-1.617
	40-49	120 (24.9%)	10 (27.0%)	130 (25.1%)	0.406	1.305	0.697-2.442
	≥ 50*	9 (1.9%)	2 (5.4%)	11 (2.1%)	0.521	1.665	0.705-1.662
Total		481 (92.9%)	37 (7.1%)	518 (100%)			

Using univariate binary logistic regression analysis OR of different risk factors were calculated.

p-value of ≤0.05 considered to be significant.

OR= Odds Ration

CI= Confidence Intervals

* Reference group

## Discussion

As our knowledge, no data is available regarding the use of EIA in detection of *C. trachomatis* antigen in women of Iran. Most of the publications on the prevalence of *Chlamydia* infection in Iran is based on serologic evaluation of antibody (8). Because IgG antibody titers persist for several months after *Chlamydia* infections, positive serological tests may indicate previous infection not active chlamydiosis. In contrast, direct Ag-EIA techniques detect specific antigens which are shed during active infections and have less cross-reactivity than serological tests detecting antibodies. On the other hands, non-invasive DNA amplification techniques are helpful since they are not dependent on viable organisms, as needed in labor-intensive culture procedures. 

In this study, positive results of EIA and all discrepant results were confirmed by nested-PCR. One sample showed a false-negative result in MOMP PCR which might be due to the presence of inhibitors in the specimen. 15 of samples showed false negative EIA results which were positive in nested-PCR. The current data shows that the sensitivity of EIA is low (59.46%) compared to MOMP-PCR (97.3%), the sensitivity of EIA is shown to be variable in different reports: from as low as 8.1% in endocervical swabs from 357 women between 16 and 41 years of age to 42.0% in endocervical swab specimens from 733 commercial sex workers, and up to 73% in a study of endocervical swabs from 109 women in Gaza (12-14). The investigation of the ability of EIA to detect *C.trachomatis* in cervical samples showed that EIA identified only 16% of specimens that contained <10 EBs by DFA staining (15). MOMP PCR showed high sensitivities in different studies, ranged from 86-98% (13, 14).

In this study all 37 positive specimens in EIA/MOMP PCR were verified by using nested-PCR and these samples were regarded as true positive, thus the specificity of the EIA and MOMP PCR was 100%. There are some studies that reported false positive results and less specificity for antigen-EIA, indicating the need for verification of positive results by other methods such as DNA amplification (16, 17). The variable performance of EIA for detection of *C.trachomatis* might be due to patient characteristics such as gender and specimen type (17, 18). One study reported the sensitivity of EIA was 73% in endocervical swabs from women and 60% in endourethral swabs from men (19). 

A systematic review of 30 studies from 1990 onward showed that pooled sensitivities for EIA was higher on endocervical swab (65%) than urine (38%) specimens (20). Study on specimens from female commercial sex workers showed that EIA sensitivity was 68.6% in vaginal swab while in endocervical swab specimens it was increased to 77.1% (21). However, some studies showed that urine is superior to other specimens for screening of *C.trachomatis* by using nucleic acid amplification tests (20, 22-24). In the current study, the overall prevalence of *Chlamydia* infection in endocervical swab samples was 7.14%. 

Several studies in other parts of Iran reported a range of 6-17% for *Chlamydia* prevalence, while other techniques and/or specimens were used (25). In a study on endocervical secretions of 123 married women with cervicitis the overall frequency of *Chlamydia* infection was 17% (21/123) (26). There are studies which investigated *C. trachomatis* infection on urine samples by PCR, one study showed 12.4% of the infertile (n=234) and 8.5% of the fertile (n=223) women were positive for *C. trachomatis* infection (8). In another study, 15% (39/130) of women were infected, while 27/130 (20.76%) were symptomatic and 12/130 (9.23%) were asymptomatic (27). Also, in report from Tehran, 12.6% of 1052 women and in a report from Sabzevar, north-east Iran, 13.77% of 196 pregnant women tested positive by PCR (28, 29). The prevalence of *Chlamydia* infection in Iranian male urine specimens was also studied that showed the rate of 10-20% in symptomatic patients when urine examined by PCR and 9.3% when cell culture was used (30-32). 

Genital *Chlamydia* infection was reported from countries neighboring Iran, which showed different prevalence rates; several of them are higher. The wide variation of *Chlamydia* prevalence rates in these studies might be due to factors, such as different population studied, socioeconomic status, and different techniques employed. In Turkey, the rates of *Chlamydia *positivity in women were ranged from less than 5.0% to more than 25% in symptomatic cases (33, 34). In Saudi Arabia, using ELISA method on endocervical/vaginal swabs, *C.trachomatis* antigen was detected in 10.5% (10/95) of pregnant women and 34.4% (35/102) of women with genital tract infection (35). In Pakistan, a national study conducted in 2004 among 730 women selling sex revealed high prevalence of STIs including 7.7% *C.trachomatis* (36). In a study in Baghdad, Iraq, *C.trachomatis* antibodies were found in 13.6% of mothers with full-term deliveries (n=198) and 6.4% of women with abortion (n=79) (37).

Similar to findings on other populations current data shows cervicitis is the most important risk factor associated with chlamydial infection of genital tracts. As shown in studies from Tehran and Lublin, Poland, women with cervicitis are more likely to have been previously infected with *Chlamydia *(14, 17, 26)*.* Urethritis and cervicitis may lead to complications including PID, ectopic pregnancy, tubal factor infertility and reactive arthritis (38). Based on the available evidence, approximately 20% of women with chlamydial lower genital tract infection will develop PID, and less proportion will develop other adverse pregnancy outcomes (39). Given that the majority of the women population in this study was in reproductive ages, genital infections acquired during pregnancy should more adversely affect maternal/neonatal morbidity. Current data showed that most of the positive cases were more than 20 years old with approximately 90% of the total chlamydiosis cases, and the majority assigned to 30-39 years age group. Similar findings were reported by other studies in married women and symptomatic/asymptomatic men from Iran (26, 30). 

Chlamydiosis should be regarded in women of reproductive ages with no genital symptoms or with symptoms such as: malodorous discharges, itching and cervicitis. 

## Conclusion

This study emphasizes the need for implementation of a routine *C. trachomatis* screening in public health programs for women particularly those with cervicitis. Since long term *C. trachomatis* infection may result in reproductive complications, early diagnosis and treatment of patients can effectively prevent adverse outcomes. The development of highly sensitive and specific nucleic acid amplification tests has improved effective diagnosis of chlamydial infections. However, molecular diagnostics need expertise and infrastructure beyond the capacities of many laboratories, hence not widely available everywhere in Iran. Primary screening of women by using a low-cost antigen based EIA is recommended, though due to the low sensitivity of EIA, verification of the negative results by a DNA amplification method is required.
